# Osteoporosis treatment prevents hip fracture similarly in both sexes: the FOCUS observational study

**DOI:** 10.1093/jbmr/zjae090

**Published:** 2024-06-11

**Authors:** Tony M Keaveny, Annette L Adams, Eric S Orwoll, Sundeep Khosla, Ethel S Siris, Michael R McClung, Mary L Bouxsein, Shireen Fatemi, David C Lee, David L Kopperdahl

**Affiliations:** Departments of Mechanical Engineering and Bioengineering, University of California, Berkeley, CA 94720, United States; Department of Research & Evaluation, Kaiser Permanente Southern California, Pasadena, CA 91101, United States; Bone and Mineral Unit, Oregon Health & Science University, Portland, OR 97239, United States; Division of Endocrinology, Kogod Center on Aging, Mayo Clinic, Rochester, MN 55905, United States; Department of Medicine, Toni Stabile Osteoporosis Center, Columbia University Medical Center, New York, NY 10032, United States; Oregon Osteoporosis Center, Portland, OR 97225, United States; Department of Orthopedic Surgery, Harvard Medical School, Center for Advanced Orthopedic Studies, Beth Israel Deaconess Medical Center, Boston, MA 02215, United States; Department of Endocrinology, Kaiser Permanente Southern California, Panorama City, CA 91402, United States; O.N. Diagnostics LLC, Berkeley, CA 94704, United States; O.N. Diagnostics LLC, Berkeley, CA 94704, United States

**Keywords:** osteoporosis, fracture risk assessment, screening, fracture prevention, antiresorptives

## Abstract

Randomized trials have not been performed, and may never be, to determine if osteoporosis treatment prevents hip fracture in men. Addressing that evidence gap, we analyzed data from an observational study of new hip fractures in a large integrated healthcare system to compare the reduction in hip fractures associated with standard-of-care osteoporosis treatment in men versus women. Sampling from 271,389 patients aged ≥ 65 who had a hip-containing CT scan during care between 2005 and 2018, we selected all who subsequently had a first hip fracture (cases) after the CT scan (start of observation) and a sex-matched equal number of randomly selected patients. From those, we analyzed all who tested positive for osteoporosis (DXA-equivalent hip BMD T-score ≤ −2.5, measured from the CT scan using VirtuOst). We defined “treated” as at least six months of any osteoporosis medication by prescription fill data during follow-up; “not-treated” was no prescription fill. Sex-specific odds ratios of hip fracture for treated vs not-treated patients were calculated by logistic regression; adjustments included age, BMD T-score, BMD-treatment interaction, BMD, race/ethnicity, and seven baseline clinical risk factors. At two-year follow-up, 33.9% of the women (750/2,211 patients) and 24.0% of the men (175/728 patients) were treated primarily with alendronate; 51.3% and 66.3%, respectively, were not-treated; and 721 and 269, respectively, had a first hip fracture since the CT scan. Odds ratio of hip fracture for treated vs not-treated was 0.26 (95% confidence interval: 0.21–0.33) for women and 0.21 (0.13–0.34) for men; the ratio of these odds ratios (men:women) was 0.81 (0.47–1.37), indicating no significant sex effect. Various sensitivity and stratified analyses confirmed these trends, including results at five-year follow-up. Given these results and considering the relevant literature, we conclude that osteoporosis treatment prevents hip fracture similarly in both sexes.

## Introduction

Osteoporosis causes over one million bone fractures annually in the USA, hip fractures being the most serious and costly.^1^^,^^2^ About one in four hip fractures occur in men,^1^ and men have higher mortality after hip fracture than women.^3^ Although the paradigm of treating women for osteoporosis to prevent fractures is well established, this is not so for men. For example, despite the availability of multiple drug treatments shown in randomized clinical trials to reduce risk of hip fracture in women,^4^ no such evidence exists for men—not because of any failed trials, but because such trials have not been conducted.^5^ Due in part to this lack of evidence, the US Preventive Services Task Force does not recommend diagnostic screening of osteoporosis for men.^6^ As a result, many men at high risk of osteoporotic fractures go untested and untreated for osteoporosis.

Despite the lack of hip-fracture clinical trial data for men, there is substantial evidence suggesting that osteoporosis treatment should be similarly effective at preventing hip fracture in both sexes. In randomized clinical trials of women at high risk for fracture, treatment-induced changes in bone mineral density (BMD) at the hip are associated with treatment efficacy for preventing hip fractures,^7^ and various osteoporosis drug treatments have been shown to increase BMD in men^8-12^ to a similar extent as in women.^13-15^ For men treated with zoledronic acid, a randomized clinical trial versus placebo demonstrated a reduction in morphometric vertebral fractures of 67%,^16^ which is similar to the 71% reported in a separate trial for women.^13^ And a meta-analysis of trials concluded that, for various (non-hip) fracture outcomes, efficacy of osteoporosis drug treatment appears to be similar between the sexes.^5^ Given that body of prior evidence, one would reasonably expect that, in patients who are at high risk of hip fracture, osteoporosis treatment should reduce the risk of hip fracture similarly in both sexes.

To address the gap in evidence of treatment effectiveness in men, we analyzed data from a large observational study of incident hip fractures in a clinical setting to compare the reduction in risk of hip fracture associated with standard-of-care osteoporosis treatment in men and women. We hypothesized that the treatment effect is similar between the sexes.

## Materials and methods

### Study design

This analysis comprised a case-control observational study of new hip fractures using existing de-identified electronic health record (EHR) information from clinical care and in which osteoporosis treatment during clinical care was the exposure. The study design was built around analyzing a patient’s existing computed tomography (CT) scan from their medical care, and thus observation started at the time of that CT scan. This analysis was part of the ongoing NIH-funded *Fracture, Osteoporosis, and CT Utilization Study* (FOCUS), originally reported in 2018^17^ and now expanded.

### Participants

The underlying patient population comprised of all Kaiser Permanente Southern California (KPSC) members aged 65 or older who had any type of abdominal or pelvic CT exam during their clinical care between January 1, 2005 and July 1, 2018 at one of 17 different medical centers. Patients were excluded if they had a hip fracture before the CT scan, any bone pathology that would exclude them from osteoporosis care, a hip implant, or had missing or otherwise unusable CT scans (see Figure S1 for details). From that population of 271,389 patients, a case-cohort sample of 11,461 patients was developed (7,913 women, 3,548 men) that comprised of all hip fracture cases from the population (within 10 years after the patient’s CT scan) as well as a randomly selected sub-sample of the population that was matched in number to the cases by sex and medical center (see Table S1 for the patient characteristic of this random sub-cohort).

**Figure 1:**
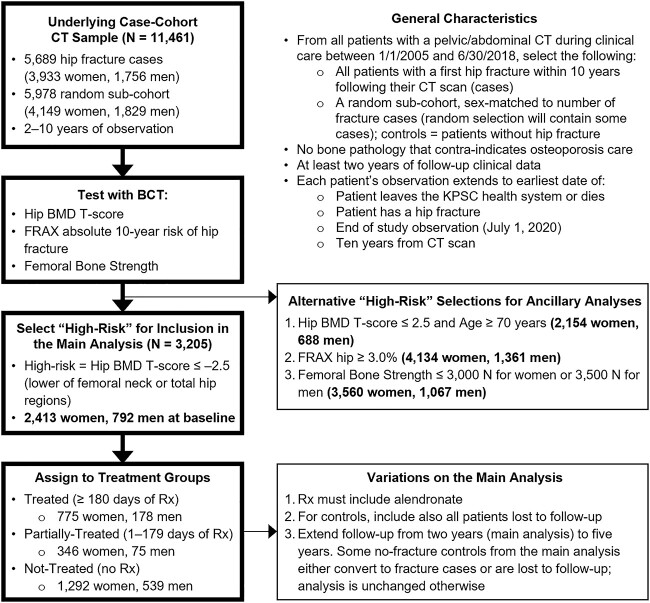
Study design and patient selection. The main analysis compared relative risk (odds ratio) of hip fracture in the treated vs not-treated patients, across the sexes, all patients being screened for BMD-defined osteoporosis at the hip (ancillary analyses screened high-risk patients in other ways, see text for details). For details of the underlying case-cohort selection, see Table S1. Rx = prescription fill for an osteoporosis medication, from patient records; for details on the types of medication, see Table S2.

Starting with this case-cohort sample, patients were then selected for inclusion in this analysis (Figure 1). Biomechanical Computed Tomography analysis (BCT) (see below) was performed on the CT scans for all the case-cohort patients to identify those at high risk of hip fracture, who were then selected for inclusion in this analysis. For our main analysis, high-risk was defined as testing positive for osteoporosis by hip BMD T-score (femoral neck or total hip T-score *≤* −2.5), a typical inclusion criterion for clinical trials of osteoporosis treatments. The resulting “analysis sample” at baseline comprised of 2,413 women and 792 men, all with BMD-confirmed osteoporosis at the hip. As described below, we also performed ancillary analyses in which we changed the definition of high-risk for inclusion, which resulted in different sample sizes (Figure 1).

### Exposure—Standard-of-care osteoporosis treatment

The “Healthy Bones Program”^18^^,^^19^ at KPSC identifies patients at high risk of fracture primarily on the basis of DXA testing, who are then typically prescribed some form of osteoporosis drug treatment following a regional protocol. In addition, these patients receive fall prevention education during a clinic visit with a nurse practitioner. This exposure was entirely independent of why the patient had a CT scan, and preceded our (retrospective) analysis and any BCT analyses. Using EHR data on prescriptions dispensed, we defined “treated,” “partially-treated,” and “not-treated” as when a patient had, respectively, at least 180 days of prescription fills of any osteoporosis medication, 1–179 days of prescription fills, or no prescription fill at all—all during the patient’s individual observation period, which started on the day of their CT scan. We did not collect information on osteoporosis treatment before the CT scan.

### Measurements and assessments

A DXA-equivalent hip BMD T-score^20-24^ (lower of the femoral neck and total hip regions; sex-specific White young-reference from NHANES) was measured from the CT scans using BCT^25^ (VirtuOst 2.4, O.N. Diagnostics). BCT was used to measure BMD because the FOCUS study design was based around the CT scan, DXA measurements were not available for all CT patients, and our previous analysis of the FOCUS cohort demonstrated that the BCT-based DXA-equivalent hip BMD T-scores predicted hip fracture equally as well as the DXA-based T-scores.^17^ We also calculated the race-specific Fracture Risk Assessment Tool (FRAX) ten-year absolute risk of hip fracture, using BMD T-scores at the femoral neck (female White young-reference for both sexes; we included all risk factors except for parental fracture history, see below) via the online FRAX calculator (Centre for Metabolic Bone Diseases, University of Sheffield). BCT was also used to measure femoral bone strength (in units of newtons, N) from finite element analysis, simulating the breaking strength of the femur for a sideways fall. Femoral strength is associated with hip fracture independently of hip BMD,^25^ and patients having low values of strength are recommended as candidates for therapeutic intervention.^26^ We also retrieved from the EHR, when available, the DXA hip and spine BMD T-scores if a DXA was taken within three years before or after the CT scan.

Data for the calculation of FRAX scores were collected from the EHR, dated to the CT scan (baseline), including age and BMI, current or recent glucocorticoid medication use (high dose prednisone or equivalent, present or past), secondary osteoporosis, smoking (≥1 smoking yr), alcohol dependence or abuse, rheumatoid arthritis, previous major (non-hip) fracture as an adult (any clinical spine fracture, forearm fracture or shoulder fracture as an adult). For seven women and three men in the analysis sample, imputed values (sex-specific mean) were used for missing data on BMI; all other data were complete.

### Statistical analysis

In our main analysis, we only included high-risk patients who tested positive for osteoporosis at the hip based on the hip BMD T-score criterion, and we followed each patient for up to two years. For that sample, we used multivariable logistic regression with hip fracture status at two years after baseline as the binary outcome to calculate the odds ratio of hip fracture for treated vs not-treated patients. This odds ratio was calculated separately for each sex. From the same logistic regression model, we also report odds ratio of hip fracture for partially-treated vs not-treated. Observation for each patient started at the CT scan and extended to the first of: (1) a hip fracture, (2) loss due to disenrollment from KPSC or death (our data did not distinguish due to patient privacy issues), or (3) two years. Any patient lost to follow-up was excluded. The multivariable model included osteoporosis treatment (3 levels) and also included variables for age, hip BMD T-score, an interaction between osteoporosis treatment and hip BMD T-score, BMI, race/ethnicity (Asian, Black, Hispanic, White or “All Other”), and all other baseline risk factors. In addition, because the random sub-cohort was matched to the cases by the medical center (*n* = 17 total), medical center was also included in the model. We also report the crude odds ratios, which had no variables in the logistic regression except for osteoporosis treatment. To test if the odds ratio for treatment differed between the sexes, we calculated the ratio of the sex-specific odds ratios (men:women) with appropriate 95% confidence intervals^27^; if these confidence intervals included unity (ratio = 1.0), we inferred no detectable sex effect.

To assess robustness of the results, two sets of ancillary analyses were performed. In the first, we varied the assumptions of the main analysis in some way: (a) we separated out treatment into one that involved alendronate (patient could also be treated with another agent) and a second that did not, and calculated odds ratio for the alendronate-involving treatment vs not-treated; (b) rather than excluding all patients lost to follow-up, we included them as part of the no-fracture controls; (c) we only included patients aged ≥ 70 years; and (d) we repeated our main analysis but extended the observation period to five years (in FOCUS, patients can have up to ten years of follow-up). In those five-year analyses, no-fracture patients from the main analysis with more than two but less than five years of follow-up were excluded, and some patients had reclassified case/control status compared to the main analysis. For example, a patient with a hip fracture occurring in year 3 would be a no-fracture control in our main analysis and a hip-fracture case in the year-5 analysis; and a no-fracture patient who passed away in year 3 would be a no-fracture control in our main analysis and would be excluded in the year-5 analysis.

In our second set of ancillary analyses, since treatment efficacy might depend on the level of fracture risk assumed for the entry criterion into the analysis,^28-30^ we changed the definition of “high-risk” for deciding which patients to include in the analysis sample; all other parameters were the same as in the main analysis. Thus, instead of defining high-risk status on the basis of hip BMD T-score by BCT, we used: (a) the FRAX score for hip fracture risk (≥ 3.0%); (b) the BCT-measured femoral bone strength (≤3000 N for women; ≤3500 N for men); (c) for the subset of patients with DXA data, we used the lowest hip BMD T-score; (d) the lowest hip or spine BMD T-score by DXA; or (e) for the hip BMD T-score by BCT, instead of using the NHANES male young-reference in the T-score calculation for the men, we used the female young-reference, as is common for clinical DXA. All statistical analyses were performed using the JMP Pro software (version 17.0.0, SAS Institute).

## Results

In the main analysis, a total of 2,413 women and 792 men were included for analysis (Table 1), all of whom had hip BMD-defined osteoporosis by their baseline CT scan. On average, mean BMD T-score at the time of the scan was 0.1 units lower for the women than men (*P* <0.0001), ranging from −3.0 to −3.2 depending on the treatment group for the women, and from −2.9 to −3.0 for the men. Median FRAX score of ten-year absolute risk of hip fracture was higher for the women (*P* <0.0001), ranging from 8.5% to 9.7% across the treatment groups compared to 6.0%–6.9% for the men. Likewise, 93.7%–95.6% of women across the treatment groups had fragile bone strength compared to 88.2%–90.7% for the men (*P* <0.0001). Thus, this was a high-risk patient population for hip fracture and the women were at slightly higher risk than the men.

**Table 1:** Baseline characteristics of the analysis sample for the main analysis, for each of the three levels of treatment (see text for details) and for both sexes.

*Characteristic*	*Women (N = 2413 total)*				*Men (N = 792 total)*				*Between sex*
	*Not-treat*	*Partial-treat*	*Treat*	*P-value* ^***a***^	*Not-treat*	*Partial-treat*	*Treat*	*P-value* ^***a***^	*P-value* ^***b***^
Number of patients	1,292	346	775		539	75	178		
Age (yr)^**c**^	82 (75–87)	82 (76–86)	80 (74–85)	<0.0001	81 (74–87)	81 (75–88)	80 (75–85)	0.80	0.19
Age ≥ 70 yr (%)	89.2	90.8	88.8	0.60	85.3	88.0	91.0	0.15	0.065
Race/ethnicity				0.032				0.86	0.15
Non-Hispanic White (%)	65.5	69.1	65.0		69.2	65.3	75.3		
Hispanic (%)	21.1	20.5	20.6		19.9	22.7	16.9		
Black (%)	6.5	6.1	4.4		5.4	5.3	3.4		
Asian or Pacific Islander (%)	5.9	3.2	8.3		4.6	5.3	3.4		
All Other (%)	1.0	1.2	1.7		0.9	1.3	1.1		
Height (m)	1.58 (0.07)	1.59 (0.07)	1.58 (0.07)	0.039	1.74 (0.08)	1.74 (0.08)	1.74 (0.08)	0.89	<0.0001
Weight (kg)	60.4 (15.3)	60.0 (14.2)	60.9 (13.7)	0.62	76.9 (17.7)	77.3 (15.1)	75.7 (14.1)	0.67	<0.0001
Body mass index (kg/m^2^)	24.0 (5.7)	23.8 (5.1)	24.5 (5.2)	0.076	25.3 (5.1)	25.6 (5.1)	25.0 (3.9)	0.65	<0.0001
Obese (BMI ≥ 30, %)	13.2	12.7	15.0	0.46	13.9	16.0	9.6	0.24	0.68
Diabetes (%)	19.9	19.9	17.0	0.24	28.2	30.7	25.3	0.63	<0.0001
Rheumatoid arthritis (%)	40.5	44.2	46.5	0.026	34.3	40.0	34.3	0.62	<0.0001
Secondary osteoporosis (%)	18.5	18.5	13.4	0.0079	20.0	13.3	13.5	0.08	0.49
Glucocorticoid use (%)	10.4	11.6	11.4	0.71	9.6	14.7	10.7	0.40	0.69
Smoker ≥1 yr (%)	20.9	22.8	19.5	0.43	32.1	34.7	28.1	0.50	<0.0001
Alcohol abuse (%)	2.4	2.3	2.1	0.88	6.7	8.0	4.5	0.48	<0.0001
Major fracture as adult (%)	20.7	23.1	24.8	0.088	12.4	17.3	23.6	0.0015	<0.0001
Hip BMD T-score^**d**^	-3.1 (0.6)	-3.2 (0.6)	-3.0 (0.5)	<0.0001	-2.9 (0.4)	-3.0 (0.4)	-2.9 (0.4)	0.42	<0.0001
Proportion ≤ -2.5 (%)	100	100	100	by design	100	100	100	by design	by design
FRAX 10-yr risk hip fracture (%)^**c**^	8.5 (5.9–13.0)	9.7 (6.3–14.0)	8.6 (5.7–13.0)	0.0032	6.0 (3.9–8.4)	6.4 (4.5–9.7)	6.9 (4.9–9.5)	0.010	<0.0001
Proportion ≥ 3.0 (%)	94.9	96.8	93.5	0.072	90.5	90.7	93.8	0.40	0.0004
Femoral strength (*N*)	2290 (470)	2260 (460)	2410 (420)	<0.0001	2890 (540)	2850 (520)	2880 (510)	0.82	<0.0001
Proportion ≤ 3000/3500 (%)^**e**^	95.6	95.1	93.7	0.16	88.5	90.7	88.2	0.84	<0.0001

Mean (SD) for continuous variables, unless noted otherwise; percentages are for categorical variables (proportional of the sample per column) unless noted otherwise. By design, all patients have osteoporosis by hip BMD T-score (either femoral neck or total hip BMD T-score ≤ −2.5). The greater number of women than men is because the number of non-fracture patients in the underlying case-cohort sample was matched one:one to the sex-specific number of hip-fracture cases and also because more women than men tested positive for osteoporosis.

a *P*-value is for the comparison of treatment groups within each sex. For continuous variables, *P*-value from one-way ANOVA except for those not normally distributed, which used the Wilcoxon test; for categorical, *P*-value is from Pearson’s test.

b *P*-value is for the comparison of average values between sex. For continuous variables, *P*-value from *t*-test (two-tail) except for those not normally distributed, which used the Wilcoxon test; for categorical, *P*-value is from Pearson’s test.

c For non-normal distributions, median and interquartile range are reported. For age, all ages above 90 years were recorded as 90 to protect patient identity; for FRAX, units are percent values.

d White sex-specific young reference NHANES for T-score calculation.

e 3,000 N for women; 3,500 N for men.

Age and race/ethnicity distributions were similar across the sexes, weight and height were lower for the women as expected, and there were differences between the sexes for most of the clinical risk factors (Table 1). Overall, all clinical risk factors differed between the sexes except for rates of secondary osteoporosis (*P* =0.49), obesity (*P* =0.68), and glucocorticoid use (*P=*0.69). Of note, the smoking rate in the men (28.1%–34.7% across the three treatment groups) was higher (*P* <0.0001) than in the women (19.5%–22.8%), and age did not differ between the sexes (*P* =0.19). Men had higher rates of diabetes (*P* <0.0001) and alcohol abuse (*P* <0.0001) than the women and lower rates of rheumatoid arthritis (*P* <0.0001). For both sexes, those treated tended to have a lower rate of secondary osteoporosis than those not-treated (*P* <0.005 women, *P* =0.08 men). The proportion of patients who had a (prior) major fracture as an adult was lower for the men than the women (*P* <0.0001), and for the men was almost two-fold lower in the not-treated than treated group (*P* =0.0015), and trended lower also for the non-treated women (*P* =0.088).

Of the 2,413 women and 792 men at baseline in the main analysis, at two years, there were 202 women (8.4%) and 64 men (8.1%) lost to follow-up and 721 women (29.9%) and 269 men (34.0%) with a hip fracture (Table 2). This proportion of fractures is lower than the approximately 50% of patients with hip fracture in the underlying case-cohort sample because fractures in the latter can occur over ten years, whereas in the main analysis, the hip-fracture cases must occur within the first two years of observation. Excluding the patients lost to follow-up, 33.9% of the women (750/2,211) and 24.0% of the men (175/728) were treated and 51.5% of the women and 66.3% of the men were not-treated (the remainder were in the partially-treated category). For the treated patients, 80.4% of the women (603/750 patients) and 89.7% of the men (157/175 patients) filled prescriptions that included alendronate; many patients had more than one type of treatment (Table S2).

**Table 2:** Number of patients for each sex at two-year follow-up by treatment status (not-treated; partially-treated; treated) and outcome status (lost to follow-up; hip fracture; no hip fracture).

		**Women**				**Men**			
		Lost	Hip FX	No FX	Total	Lost	Hip FX	No FX	Total
Not-treated	*N*	154	475	663	1,292	56	212	271	539
	%	76.2	65.9	44.5	53.5	87.5	78.8	59.0	68.1
Partially-treated	*N*	23	134	189	346	5	28	42	75
	%	11.4	18.6	12.7	14.3	7.8	10.4	9.2	9.5
Treated	*N*	25	112	638	775	3	29	146	178
	%	12.4	15.5	42.8	32.1	4.7	10.8	31.8	22.5
Total	*N*	202	721	1,490	2,413	64	269	459	792

*N* = number of patients at baseline; percentage values refer to each column’s total.

Results from the logistic regression analyses indicated that the odds ratio of hip fracture associated with osteoporosis treatment demonstrated a substantial reduction in risk, which did not differ between the sexes (Table 3). For treated vs not-treated patients, the adjusted odds ratio of hip fracture was 0.26 (95% confidence interval: 0.21–0.33) for women and 0.21 (0.13–0.34) for men. The ratio of adjusted odds ratios (men:women) was 0.81 (0.47–1.37), indicating no significant sex effect. As expected, the odds ratios were consistently higher (smaller treatment effect) for the partially treated patients (vs not-treated); these odds ratios were not significant for either sex and did not depend on sex. The crude odds ratios were the same between the sexes—0.25 (0.19–0.31) for women vs 0.25 (0.16–0.39) for men—and were similar to the adjusted values, indicating that the multiple covariates in the adjusted models had a minimal effect on how osteoporosis treatment was associated with risk of hip fracture.

**Table 3:** Odds ratio (adjusted and crude) of hip fracture associated with osteoporosis treatment (treated vs not-treated patients; and partially-treated vs not-treated patients) for each sex at two-year follow-up.

			**Adjusted odds ratio (95% CI)**		**Crude odds ratio (95% CI)**	
**Treated vs not-treated**						
		Women	0.26	0.21–0.33	0.25	0.19–0.31
		Men	0.21	0.13–0.34	0.25	0.16–0.39
		Men:Women^a^	0.81	0.47–1.37	1.00	0.60–1.66
**Partially-treated vs not-treated**						
		Women	0.90	0.69–1.18	0.99	0.77–1.27
		Men	0.69	0.40–1.21	0.85	0.51–1.42
		Men:Women^a^	0.77	0.41–1.42	0.86	0.49–1.52

The men:women ratio of odds ratios statistically compares the sex-specific odds ratios.

Sample size = 2,211 women and 728 men; analysis excludes any patients lost to follow-up. Ranges show the 95% confidence intervals. All logistic regression models included osteoporosis treatment as a variable. Adjusted models also included age, BMI, race/ethnicity, imaging facility, all baseline clinical risk factors (see text), hip BMD (lower BMD T-score of the femoral neck or total hip regions), and an interaction term between BMD T-score and osteoporosis treatment; the crude model has no extra variables.

a If this 95% confidence interval includes a value of 1.00, the odds ratios do not significantly differ between the sexes.

Further analyses of the crude odds ratio revealed that the risk (odds) of hip fracture for treated vs not-treated patients was similar between the sexes because the risk of fracture for treated patients was similar between the sexes, as was the risk of fracture for untreated patients. Mathematically, the crude odds ratio from logistic regression for our study design equals the crude odds ratio by simple head count. As can be calculated from the head-count data (Table 2), for the treated patients, the odds of hip fracture was similar for both sexes (women: 0.176 = 112 fracture/638 no-fracture; men: 0.199 = 29/146), and for the not-treated patients, the odds of hip fracture was also similar for both sexes (women: 0.716 = 475/663; men: 0.782 = 212/271). As a result, the (crude) odds ratio of hip fracture for treated vs not-treated patients was the same for both sexes (women: 0.25 = 0.176/0.716; men: 0.25 = 0.199/0.782).

**Figure 2:**
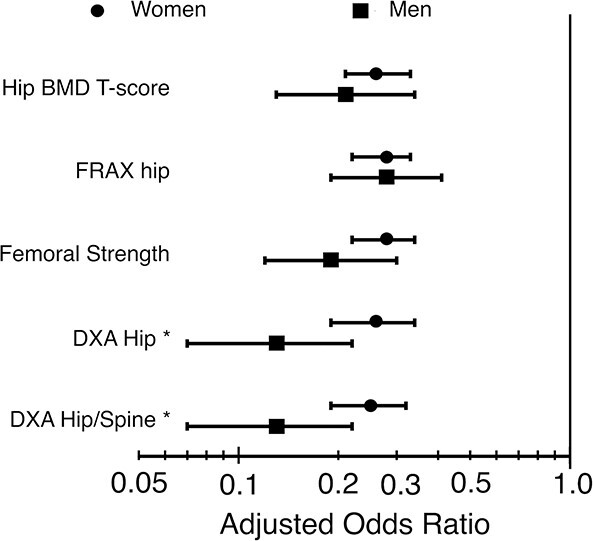
Adjusted odds ratio of hip fracture associated with osteoporosis treatment for each sex. Labels on the left show the criteria by which patients at high-risk of hip fracture were included in the analysis (see text for details), the top row being the main analysis. Data are shown at year two of follow-up. Error bars show 95% confidence intervals. Lower values of odds ratios denote a larger treatment effect. ^*^ DXA was performed within three years before or after the CT scan and was not available for all patients in the underlying case-cohort sample. Odds ratios by DXA were significantly lower for men than women (*P* <0.05 at least).

The two sets of ancillary analyses indicated that these trends remained robust for all scenarios. The adjusted odds ratios for each sex changed little and remained similar between the sexes when treatment was restricted to include alendronate, when only those aged ≥70 years were included, when the observation period from baseline was extended out to five years, and when we included all lost patients in the analysis as additional no-fracture controls (Table 4). Changing how we defined “high-risk” for including patients in the analysis also did not change the trends (Figure 2). Interestingly, when the FRAX hip score (≥3.0%) was used instead as the criterion to include patients, the adjusted odds ratio for treatment was the exact same between the sexes (0.28, 0.23–0.34 women; 0.28, 0.19–0.41 men). Also, when we included patients instead based on available DXA measurements from clinical practice (within three years before or after the CT scan), the treatment effect size barely changed for the women but became stronger for the men, resulting in a sex difference in the odds ratio (ratio of odds ratios for men:women = 0.50, 0.26–0.95 for hip DXA; 0.52, 0.28–0.98 hip/spine DXA). Part of that effect was due to DXA using a female young-reference for the T-score calculation, which strengthened the treatment effect for men when applied to the BCT-based hip BMD T-score (odds ratio 0.21 using male young reference, as in the main analysis; odds ratio of 0.18 using female young reference).

**Table 4:** Adjusted odds ratio for variations of the main analysis.

		**Sample size**	**Odds ratio**	**95% CI**
***Main analysis (reference)***				
	Women	2,211	0.26	0.21–0.33
	Men	728	0.21	0.13–0.34
	Men:Women^a^		0.81	0.47–1.37
**Variations on the main analysis:**				
*Treatment must include ALN*				
	Women	2,211	0.24	0.18–0.31
	Men	728	0.21	0.13–0.34
	Men:Women^a^		0.88	0.50–1.52
*Assume drop-out = no fracture and include*				
	Women	2,413	0.31	0.25–0.40
	Men	792	0.26	0.16–0.41
	Men:Women^a^		0.84	0.50–1.42
*Only include age ≥ 70 yr*				
	Women	1,980	0.27	0.21–0.34
	Men	633	0.20	0.12–0.32
	Men:Women^a^		0.74	0.43–1.28
*Extend the follow-up observation to five years*				
	Women	1,985	0.43	0.34–0.54
	Men	680	0.34	0.21–0.53
	Men:Women^a^		0.79	0.47–1.33

In all analyses, the same covariates were used in the multivariable logistic regression model as in the main analysis. See text for details. ^a^If this 95% confidence interval includes a value of 1.00, the odds ratios do not significantly differ between the sexes.

ALN = the treatment included alendronate but could have also included additional treatments. See Table S2 for details on treatment types.

## Discussion

For men, no randomized clinical trial has been performed for osteoporosis drug treatment with hip fracture as an adequately powered outcome, and none may ever be done due to the required expense. Addressing that evidence gap, we found that in this patient population—which was confirmed to be at high risk of hip fracture by multiple different metrics (BMD, FRAX, and femoral bone strength)—the reduction in risk of hip fracture associated with standard-of-care osteoporosis treatment was at least as large for men as for women. Including hundreds of hip-fracture cases, this is perhaps the largest ever study of treatment effects on risk of hip fracture, and the only one to directly compare treatment effects between the sexes. Given its setting, our results reflect actual clinical practice. The accumulated evidence^5^^,^^7-16^ has previously suggested that osteoporosis treatment should reduce hip fractures similarly in both sexes, and this expectation was unambiguously confirmed by our results. Thus, given our new results and that prior evidence, we conclude that osteoporosis treatment prevents hip fracture similarly in both sexes.

Our results indicate that the overall treatment effect was similar between the sexes for this high-risk patient population because the risk of hip fracture was similar between the sexes both for the treated and not-treated patients. Reflecting what is well known, there were more hip fractures for the women than men in our underlying patient population and case-control cohort. That epidemiology mostly reflects that more women than men in the general population are at high risk of hip fracture, as in the random selection of our underlying patient population (18.0% women vs 10.3% men by hip BMD T-score; 34.6% vs 20% by FRAX hip score; and 30.7% vs 16.1% by femoral bone strength; see Table S1). Our new results showed that when a patient is confirmed to be at high risk of hip fracture—by either BMD T-score, FRAX hip score, or femoral strength criteria—their risk (odds) of fracture did not depend on sex, and this was true both for treated and untreated patients. Because of that, the treatment effect was similar between the sexes. This sex-independent treatment effect is consistent with the previous literature, which has shown that treatment-induced changes in BMD at the hip for women are associated with treatment efficacy for preventing hip fractures^7^ and that treatment-related increases in BMD for women^13-15^ are similar to those in men.^8-12^ Mechanistically, these collective findings suggest that for patients at high risk of hip fracture, the drug treatment efficacy is dominated by treatment-induced changes in the bone that do not depend on sex.

Although we did not perform a randomized clinical trial, which is the gold standard for establishing causation and absolute effect size of any observed treatment effects, our study design was nonetheless valid for addressing our objective of comparing treatment effects between the sexes. With our study design, biases could exist in the patient selection and the exposure. Our underlying patient population was comprised of patients in a large healthcare system undergoing pelvic/abdominal CT for any reason during their medical care. The medical indications for such a CT exam are not related to osteoporosis status and thus this element of the patient selection is unlikely to have introduced any relevant bias between the sexes. Similarly, there was no sex bias in any of our exclusion criteria (see Figure S1). As is typical in a clinical trial, we excluded patients with a bone pathology that would contraindicate an osteoporosis assessment and those with a prior hip fracture or hip implant. We then “enrolled” patients for analysis who tested positive for being at high risk of hip fracture via hip BMD T-score criteria, again, typical of a clinical trial and the same criterion applied equally to both sexes. Because fewer men than women are at high risk of hip fracture or suffer hip fractures, our analysis cohort had fewer men than women. But that imbalance has no effect on calculation of odds ratios associated with treatment for either sex, and thus was not a source of bias. Likewise, the odds ratios for treatment do not depend on the selected number of hip-fracture cases and no-fracture controls in the sample; those numbers only affect generality and confidence intervals.

Any biases associated with the exposure may affect the magnitude of the odds ratios reported here but are unlikely to favor either sex and thus should not affect our comparison between the sexes. The exposure was osteoporosis treatment via prescription records after the CT scan, with the prescription being issued at KPSC primarily on the basis of DXA testing at some point during the patient’s medical care. It is possible that some of the men and women in our study were selected for treatment on slightly different bases (for example, routine DXA testing at KPSC typically occurs slightly later for the men; and some men may be reluctant to take a DXA or a prescribed osteoporosis medication, thinking that osteoporosis is not important for men). Indeed, the treatment rate for the men analyzed in our study (24.0%) was lower than for the women (33.9%). A lower treatment rate per se will not affect the odds ratio for treatment for either sex. However, for the men, we did observe a slightly lower baseline risk profile (by median FRAX hip score) for the non-treated than treated groups, likely perhaps due to their two-fold lower (*P* =0.0015) rate of previous fracture as an adult. That effect was much smaller for the women (Table 1). If anything, these traits for the men might make the drug look worse because one would expect fewer new hip fractures in the non-treated men since they are at lower risk. Since we observed an opposite trend, this possible bias was presumably unimportant. Another possibility for this study, reflecting real-world clinical practice, is that some high-risk patients had an increased degree of immobility due to whatever illness or condition required their CT scan. That immobility could reduce their risk of falling, protecting them from hip fracture. Such a mobility effect would make the drug look better if the effect was mostly in treated patients, worse if mostly in untreated patients, and would wash out if similar in both treated and untreated patients. Importantly, while such a possible bias could affect the magnitude of the odds ratio associated with treatment for each sex, this bias is unlikely to manifest itself differently between the sexes. Likewise, any other biases for this study design relevant to risk of hip fracture, including any possible bias by indication, are unlikely to have differed appreciably between the sexes. This is in large part because we already screened all patients for inclusion to be at high-risk of fracture, and thus any residual between-sex bias should only have minor effects on the risk profile.

Supporting the general validity of our analysis, our reported odds ratios in women are consistent with what has been observed in comparable randomized control trials. In the “FIT” randomized clinical trial for alendronate vs placebo in post-menopausal women, the cumulative relative risk of hip fracture was 0.35 (95% confidence intervals ~0.15–0.71) at the two-year follow-up.^31^ Because hip fracture is a rare event, mathematically, an odds ratio based on the two-year outcomes should approximate a cumulative relative risk at two years and thus one can compare the two metrics. We found an odds ratio of 0.24 (0.18–0.31) for women when we required treatment to include alendronate, which is numerically lower but statistically consistent with the FIT cumulative relative risk (the calculated ratio of FIT/FOCUS relative risks is 1.46 (95% CI: 0.64–3.32), indicating no significant difference between the two). Consistent with our finding in FOCUS that the adjusted and crude odds ratios were similar, indicating a negligible role of the various clinical risk factors on the treatment effect, this consistency between the FOCUS and FIT values of relative risk persisted despite substantial differences between the two cohorts: overall the volunteering participants in FIT were presumably healthier than patients having CT in routine clinical care; and the women in FIT were over ten years younger than in our study.

Given its observational nature, our study has a number of additional limitations and caveats, but none of which should compromise our conclusions. First, our underlying patient population was selected on the basis of having had a hip-containing CT scan for some medical purpose, perhaps indicating a different risk profile than the general population, in turn which might influence the absolute efficacy of drug treatment. As discussed above, that effect should be small and should not affect the between-sex comparison. Second, we did not collect data for any prior osteoporosis treatment before the patient’s CT scan. Any prior treatment before the baseline CT scan for some of the non-treated patients—a “drug holiday”—might make the drug look worse since there would be fewer fractures in our not-treated group for such drug-holiday patients. If more women than men at KPSC are on drug holidays, then this could explain why some of the odds ratios trended numerically higher (worse) for the women. One would expect any protective effect of drug holidays in the non-treated patients to diminish over time, which would be associated with an improving (decreasing) odds ratio over time. Our finding of similar odds ratios at the two-year and five-year observations, for both sexes, therefore suggests that any such prior-treatment effects were minor.

An additional caveat is that our input for the FRAX calculation did not include parental history of fracture. As a result, our reported FRAX scores may underestimate the true absolute ten-year risk of hip fracture. However, any such underestimation should be similar for both sexes and therefore should not compromise our between-sex comparisons. FRAX was used in this study as an alternate method to identify “high risk” patients; we did not address its accuracy for assessing ten-year risk for either sex in this cohort, which was not relevant to our objectives. Interestingly, when high-risk status for inclusion in the analysis was defined by the FRAX hip score instead of the hip BMD T-score, the odds ratio for treatment was the exact same for both sexes. That finding suggests that in selecting patients for treatment, efficacy might be more determined by level of fracture risk than by BMD status. The still-large effect size with inclusion by FRAX (the odds ratio was 0.28) also indicates that many patients with osteopenia (low bone mass) at the hip benefited from treatment. For example, the high-risk FRAX cohort was substantially larger than the high-risk BMD cohort (5,495 vs 3,205 patients, see Figure 1) and had 42% of patients without BMD-defined hip osteoporosis; similarly, the high-risk femoral bone strength cohort had 31% of patients without BMD-defined hip osteoporosis. Finally, in calculating the odds ratios, we did not account for any competing risk of death — in our main analysis all patients who died or left the Kaiser healthcare system (we could not differentiate) before two years were excluded from the logistic regression analysis. That loss rate was similar between the sexes (8.4% vs 8.1%), suggesting no bias between the sexes. Further, in our ancillary analysis that instead included these lost-to-follow-up patients as no-fracture controls, the odds ratios only increased slightly (0.31 vs 0.26 women; 0.26 vs 0.21 men). Thus, although he absolute effect size for treatment reported here could change with alternative statistical modeling that accounted for competing risk of death, omitting this detail is unlikely to have biased our comparison between the sexes and thus should not affect our overall conclusion.

Clinically, these results should have implications for how men are managed for osteoporosis. In 2018, the *US Preventive Services Task Force* concluded that the evidence was insufficient to assess the balance of benefits and harms of screening for osteoporosis to prevent osteoporotic fractures in men.^6^ More recently, the *American College of Physicians* recommended that clinicians offer treatment to men at high risk for fracture, but this was presented as a “conditional recommendation” with “low-certainty evidence”.^32^ We did not address cost-effectiveness of diagnostic screening and treatment for men, nor what is the optimal way to define “high-risk” for initiating treatment for either sex. Nonetheless, our study does provide more conclusive evidence, particularly when also considering the accumulated literature,^5^^,^^8–15^^,^^33^ that osteoporosis treatment in clinical practice prevents hip fractures similarly in high-risk men and women, whether “high-risk” is defined by DXA, FRAX, or BCT. Given the devasting effects of hip fracture, especially for men,^1^^,^^3^ clinical guidelines for osteoporosis testing and treatment should be updated to reflect this new evidence.

## Supplementary Material

OPDrug_Supplementary_Material_zjae090

## Data Availability

Data may be available upon request from the authors. The data that support the findings of this study are available from the corresponding author upon reasonable request for academic studies and are not publicly available due to privacy, ethical, and/or proprietary restrictions.
