# Supplementary material for: Osteoporosis treatment prevents hip fracture similarly in both sexes: the FOCUS observational study
Source: J Bone Miner Res. 2024 Jun 11;39(10):1424–33. doi: 10.1093/jbmr/zjae090 (PMC11425693; doi:10.1093/jbmr/zjae090)
Supplement: OPDrug_Supplementary_Material_zjae090 [file opdrug_supplementary_material_zjae090.pdf]

SUPPLEMENTARY MATERIALS

The underlying patient population (**Supplemental Figure S1**) comprised all KPSC members aged 65 or older who had any type of abdominal or pelvic CT exam between January 1, 2005 and July 1, 2018. Eligible patients were identified using EHR demographic information and ICD-9/ICD-10 diagnostic and procedure codes, including ICD-9/ICD-10 diagnosis codes for inpatient hospitalizations for any hip fractures. Each patient's observation period started on the date of their CT exam and ended at the earliest of a hip fracture, disenrollment from KPSC, death, 10 years follow up, or the end of data collection (December 31, 2020), whichever came first. Patients were excluded if they: 1) were enrolled in KPSC for less than one year before the CT exam; 2) had any type of hip fracture before the CT exam or a high-energy hip fracture after the CT exam; 3) had a metal implant at the hip; 4) had a diagnosis of an excluded bone pathology (malignant neoplasm of the femur, multiple myeloma, osteomalacia, hypophosphatasia, Paget's disease, osteogenesis imperfecta, or osteopetrosis); 5) were missing information on the CT imaging facility; or 6) had been excluded from the original FOCUS due to a non-usable CT scan. Of the 341,364 patients initially identified, the 271,389 patients (54% women) who met the inclusion and exclusion criteria comprised our source population.

This source population was then used to construct a case-cohort study sample. Cases were all those in the source population who had a first (fragility) hip fracture during their observation period. A sub-cohort was then selected via random sampling of the source population (including all cases), matching the cases by the CT imaging facility (n=17 total) and providing an approximate 1:1 ratio of cases to non-cases for each sex. After combining the cases with the sub-cohort and discarding ineligible patients (primarily with incomplete, inaccessible, or unusable imaging data), the resulting analysis sample comprised of 11,461 patients who had complete data (7,913 women, 3,548 men), approximately half with hip fracture.

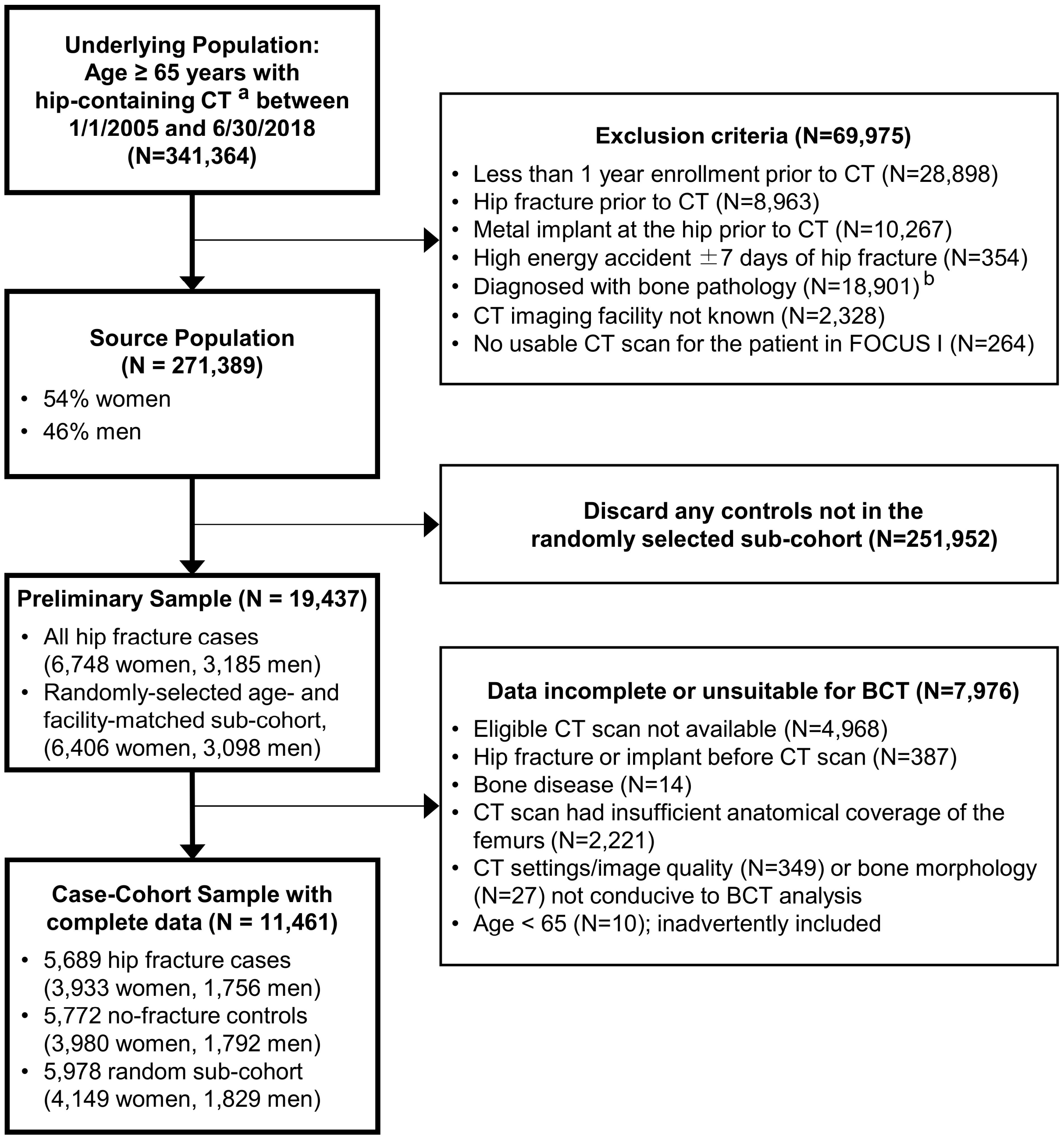

Supplemental Figure S1: Patient population and the underlying case-cohort sample.

Supplemental Table S1: Characteristics of the underlying patient population at baseline, based on the random sub-cohort, comparing both sexes. By design, the population was age-matched between the sexes. The greater number of women in the random sub-cohort is because it approximately matched the female:male ratio of hip fractures in the patient population.

Characteristic	*Women*	*Men*	*p-value †*
Number of patients	4149	1829	
Age (years) *	72 (67–78.5)	72 (68–79)	0.71
Age range (years) **	65–90	65–90	
Age ≥ 70 years (%)	62.0	61.2	0.58
Race/ethnicity			0.002
Non-Hispanic White (%)	52.5	56.0	
Hispanic (%)	26.5	24.8	
Black (%)	11.4	8.5	
Asian or Pacific Islander (%)	8.5	9.3	
All Other (%)	1.1	1.5	
Height (m)	1.60 (0.07)	1.74 (0.08)	< 0.0001
Weight (kg)	70.9 (17.4)	83.7 (17.1)	< 0.0001
Body mass index (kg/m^2) ***	27.8 (6.3)	27.6 (5.0)	0.26
Obese (BMI ≥ 30, %) ***	30.4	26.3	0.002
Diabetes (%)	26.3	29.9	0.004
Rheumatoid arthritis (%)	39.3	27.6	< 0.0001
Secondary osteoporosis (%)	11.4	12.9	0.098
Glucocorticoid use (%)	11.8	7.6	< 0.0001
Smoker ≥ 1 year (%)	18.6	29.4	< 0.0001
Alcohol abuse (%)	1.4	4.5	< 0.0001
Major fracture as adult (%)	8.6	3.9	< 0.0001
Lowest hip BMD T-score	-1.5 (1.1)	-0.7 (1.2)	< 0.0001
High Risk (%)	18.0	10.3	< 0.0001
FRAX 10-year risk hip fracture (%) *	1.7 (0.7–4.2)	1.1 (0.5–2.5)	< 0.0001
High Risk (%)	34.6	20.0	< 0.0001
Femoral strength (N)	3460 (860)	4570 (1120)	< 0.0001
High Risk (%)	30.7	16.1	< 0.0001

Mean ± SD for continuous variables, unless noted otherwise; percentages for categorical variables.
† for continuous variables, p-value from t-test (two-tail) except for those not normally distributed, which used the Wilcoxon test; for categorical, p-value is from Pearson's test.
* Not normally distributed, therefore median and interquartile range are reported.
** All ages above 90 years were recorded as 90 years to protect patient identity.
*** BMI data were not available for 26 women and 15 men.

Supplemental Table S2: Number of high-risk patients (sex-specific hip BMD T-score ≤ -2.5) available at baseline and two-year follow up and taking particular treatments between baseline and follow-up.

	Women	Men
Total Patients at Baseline	2275	725
Drop Out: Exit KPSC or Death	194 (8.5%)	61 (8.4%)
*Total Patients at Follow-Up **	2081	664
Not-Treated	1068 (51.3%)	440 (66.3%)
Partially-Treated	302 (14.5%)	60 (9.0%)
Treated	711 (34.2%)	164 (24.7%)
*Treatment Type ***		
alendronate	566 [216]	145 [50]
calcitonin	122 [71]	24 [8]
denosumab	38 [2]	2 [0]
etidronate	4 [1]	1 [0]
ibandronate	40 [12]	4 [1]
raloxifene	28 [3]	0 [0]
risedronate	29 [12]	7 [3]
teriparatide	7 [3]	1 [0]
zoledronic acid	60 [9]	9 [0]

Number of high-risk patients (sex-specific hip BMD T-score ≤ -2.5) at baseline, follow up, and taking particular treatments between baseline and follow-up. Total number of patients decreases over time due to non-fracturing patients dropping out (either dying or leaving the KPSC system). Total patients by treatment type exceeds the number of patients treated because some patients took more than one type of treatment.

* Treatment means ≥ 180 days of prescription fills for an osteoporosis medication during the observation period; partially-treated means between 1–179 days of prescription fills during the observation period; not-treated means no prescription fill at all during the observation period.

** Values are for treated patients at follow-up. Parenthetical values are for partially-treated patients.
